# Do Orally Disintegrating Tablets Facilitate Medical Adherence and Clinical Outcomes in Patients with Post-stroke Dysphagia?

**DOI:** 10.1007/s00455-024-10737-8

**Published:** 2024-08-03

**Authors:** So Sato, Yusuke Sasabuchi, Akira Okada, Hideo Yasunaga

**Affiliations:** 1 Department of Clinical Epidemiology and Health Economics, Graduate School of Medicine, The University of Tokyo, 7-3-1, Hongo, Bunkyo, Tokyo 1130033 Japan; 2 The Department of Real-World Evidence, Graduate School of Medicine, The University of Tokyo, Bunkyo, Japan; 3 Department of Prevention of Diabetes and Lifestyle-Related Diseases, Graduate School of Medicine, The University of Tokyo, Bunkyo, Japan

**Keywords:** Deglutition disorders, Medication adherence, Propensity score, Stroke

## Abstract

Orally disintegrating tablets (ODTs) dissolve rapidly in contact with saliva and have been reported to facilitate oral administration of medications in swallowing difficulties. However, their clinical benefits remain unclear because no previous studies have examined whether ODTs facilitate medication adherence and clinical outcomes in patients with post-stroke dysphagia. This study evaluated the association between ODT prescriptions and clinical benefits using high-dimensional propensity score (hd-PS) matching to adjust for confounding factors. Using a large Japanese commercial medical and dental claims database, we identified patients aged ≥ 65 years with post-stroke dysphagia between April 2014 and March 2021. To compare 1-year outcomes of medication adherence, cardiovascular events, and aspiration pneumonia between patients taking ODTs and non-ODTs, we performed hd-PS matching. We identified 11,813 patients without ODTs and 3178 patients with ODTs. After hd-PS matching, 2246 pairs were generated. Medication adherence for 1 year, based on the proportion of days covered, was not significantly different between the non-ODT and ODT groups before (0.887 vs. 0.900, P = 0.999) and after hd-PS matching (0.889 vs. 0.902, P = 0.977). The proportion of cardiovascular events (0.898 vs. 0.893, P = 0.591) and aspiration pneumonia (0.380 vs. 0.372, P = 0.558) were also not significantly different between the groups. This study found no significant differences in medication adherence, cardiovascular diseases, or aspiration pneumonia between the non-ODT and ODT groups in patients with post-stroke dysphagia. Both groups achieved a proportion of days covered exceeding 80%. Clinicians may consider prescribing ODTs or non-ODTs based on patient preferences rather than solely on post-stroke conditions.

**Supplementary Information:**

The online version contains supplementary material available at 10.1007/s00455-024-10737-8.

## Introduction

Orally disintegrating tablets (ODTs) disintegrate or dissolve rapidly in the oral cavity upon contact with saliva [1]. Patients do not need to chew or drink additional water when they take ODTs, and ODTs facilitate oral administration for those with difficulty swallowing [2–5]. Because ODTs can be easily taken by patients with difficulty swallowing, they are expected to improve medication adherence [1]. A previous review reported that at least 37–45% of post-stroke patients had dysphagia due to malfunction of the swallowing mechanism [6]. Therefore, ODTs may be a promising treatment option for patients with post-stroke dysphagia.

In a previous study, patients with dysphagia caused by stroke or cancer taking ODTs showed better swallowing performance than those taking non-ODTs, as evaluated by endoscopy and electromyography, compared to those taking non-ODTs [5]. Another study reported that 28% of diabetic patients improved their medication adherence after switching from non-ODTs to ODTs [7].

However, the benefits of ODTs in patients with post-stroke dysphagia remain unclear. A previous study reported no differences in residuals in the pharynx and larynx or in aspiration between ODTs and non-ODTs [5]. Moreover, to the best of our knowledge, no previous studies have reported the clinical benefits of ODTs in patients with post-stroke dysphagia. Therefore, the present study aimed to evaluate the effect of ODTs on medication adherence and clinical outcomes in post-stroke patients with dysphagia compared with non-ODTs.

## Materials and Methods

### Data Source

This study utilized the DeSC database (DeSC Healthcare, Inc.), a large commercial medical and dental claims database in Japan. Studies using this database can be found elsewhere [8, 9]. This database contains health insurance claims data from multiple types of health insurers: (i) National Health Insurance for individual proprietors and unemployees (Kokuho), (ii) health insurance for employees of large companies (Kempo), and (iii) the Advanced Elderly Medical Service System for those aged 75 years and over (Koki Koreisha Iryo Seido). Thus, the DeSC database included young, middle-aged, and elderly individuals. Medical and dental claims data for outpatients and inpatients were anonymized at the individual level. The database includes the following information: (i) unique identifier, (ii) age and sex, (iii) diagnoses based on the International Classification of Diseases, 10th Revision (ICD-10) codes, (iv) procedures, (v) drugs dispensed based on the Anatomical Therapeutic Chemical Classification System, and (vi) dates of enrollment and disenrollment in insurance. The DeSC database contains information on approximately 12,000,000 individuals, and its age distribution of the DeSC database is comparable to the Japanese population estimates [9].

### Study Design and Patient Selection

This retrospective cohort study used the data collected between April 2014 and March 2021. A new prevalent user design was used [10]. We included patients who (i) were diagnosed with stroke (ICD-10 I60–I63), (ii) underwent swallowing rehabilitation within a year from the diagnosis of stroke, (iii) were newly prescribed the target drugs listed in Online Resource 1 (initial pharmacotherapy for hypertension, diabetes mellitus, or hyperlipidemia), and (iv) had at least 1 year of any records in the DeSC database before cohort entry. We defined the patients’ age on the day of new use of the target drugs. We excluded patients who (i) were prescribed the target drugs within the previous year, (ii) were newly prescribed the target ODTs and non-ODTs at the same time, (iii) were under 65 years of age since those aged ≥ 65 years were reportedly more likely to experience dysphagia than the younger age groups [11], (iv) were prescribed the target drugs less than twice in the study period, and (v) had a gastrostomy before cohort entry. The patients were followed-up from the initiation of the target drugs to assess the outcomes.

### Exposure of Interest

We compared patients who received targeted ODTs (ODT group) with those who received active comparators (non-ODT group). Target ODTs were defined as medications used for hypertension, diabetes mellitus, and hyperlipidemia (Online Resource 1). This is because the Japanese Guidelines for the Management of Stroke 2021 recommend medication to prevent recurrent strokes (grades A–C) [12]. Active comparators (non-ODTs) included all drugs with the same target disease as the ODTs.

### Variables

We included the following variables in the model: age (65–74, ≥ 75 years), sex, diagnoses, procedures, and drug use history during the pre-prescription period. The variables included in the analyses were based on previous studies investigating aspiration, dysphagia, and salivary disorders [6, 13, 14]. Further details of these variables are provided in Online Resource 2. In this study, the pre-prescription period was defined as the period within 365 days of the initial use of ODTs or active comparators.

### Outcome Measurements

The primary outcome was the target drug proportion of days covered (PDC) for 1 year as the patient’s medication adherence [15]. We defined the start date as the first fill date for each target drug and the end date as the date of the last fill. The evaluation period for PDC was set at 1 year, which is the minimum recommended evaluation period according to the guidelines of the National Association of Specialty Pharmacy Clinical Outcomes Committee Adherence Workgroup and the American Society of Health-System Pharmacists section of specialty pharmacy practitioner outcomes and value section advisory group [15]. If the follow-up period was less than 1 year, the PDC for the observed period was assumed to continue for 1 year. We defined the medication adherence threshold as a PDC of 80%, following a previous study of medication adherence [16]. The secondary outcomes were hospital admission for aspiration pneumonia (ICD-10: J69), cardiovascular events (i.e., heart failure (ICD-10: I50, I11), atrial fibrillation (ICD-10: I48), myocardial infarction (ICD-10: I21), angina pectoris (ICD-10: I20), stroke (ICD-10: I60–63), and composite event of cardiovascular diseases [17] within 1 year as a benefit of medication adherence.

### Statistical Analysis

#### Hd-PS Estimation

In this study, we used a large claims database in Japan to obtain a large study sample. We conducted a high-dimensional propensity score (hd-PS) matching analysis to balance patient backgrounds and account for confounding factors. Hd-PS analysis was proposed for studies using administrative claims databases to improve the ability to control confounding factors in comparative effectiveness [18]. We performed hd-PS matching between ODT and non-ODT groups. The detailed methods for hd-PS estimation have been described previously [19]. Briefly, the following five processes were conducted: (i) definition of different data dimensions; (ii) identification of empirical candidate covariates in each data dimension during the pre-prescription period; (iii) assessment of the frequencies of candidate covariates; (iv) ranking of candidate covariates of all dimensions by their potential as confounding factors based on Bross’s formula [20], and (v) selection of the top *n* potential covariates for propensity score modeling.

We structured the following ten dimensions: medical outpatient diagnoses, medical inpatient diagnoses, dental outpatient diagnoses, dental inpatient diagnoses, medical outpatient procedures, medical inpatient procedures, dental outpatient procedures, dental inpatient procedures, and outpatient and inpatient drug use. We included the 500 highest potential covariates and variables described in Online Resource 2 in the logistic regression model for receiving the target ODTs to estimate hd-PS.

#### Propensity Score Matching

We performed 1:1 nearest-neighbor matching with the caliper width set at 20% of the standard deviation of the propensity score. The balancing properties of the matching covariates were examined using absolute standardized differences between the groups. An absolute standardized difference of > 10% was considered to indicate an imbalance. The chi-squared test was used for categorical variables and Student’s t-test was used for continuous variables to compare the outcomes between the ODT and non-ODT groups.

#### Subgroup Analysis

We conducted subgroup analyses stratified by treatment with ODTs (hypertension, diabetes, and hyperlipidemia) to compare medication adherence between the ODT and non-ODT groups. Subgroup analyses were also performed, stratified by the presence of polypharmacy (non-polypharmacy, polypharmacy, and hyperpolypharmacy). All prescriptions were identified to determine the number of drugs administered by each patient. Patients were considered to have taken prescribed medications within 60 days prior to cohort entry [10]. We calculated the number of drug classes taken by each patient from the 86 classes (Online Resource 3). Based on the number of prescribed drugs, we classified the patients into three groups [21]: no polypharmacy, polypharmacy (five to nine classes), and hyper-polypharmacy (ten or more classes).

The threshold for significance was set at P = 0.05. The hd-PS estimation was performed using R version 3. 6. 1. (R Foundation for Statistical Computing, Vienna, Austria). The remaining statistical analyses were performed using Stata SE (version 17.0; StataCorp, College Station, TX, USA).

## Results

**Fig. 1:**
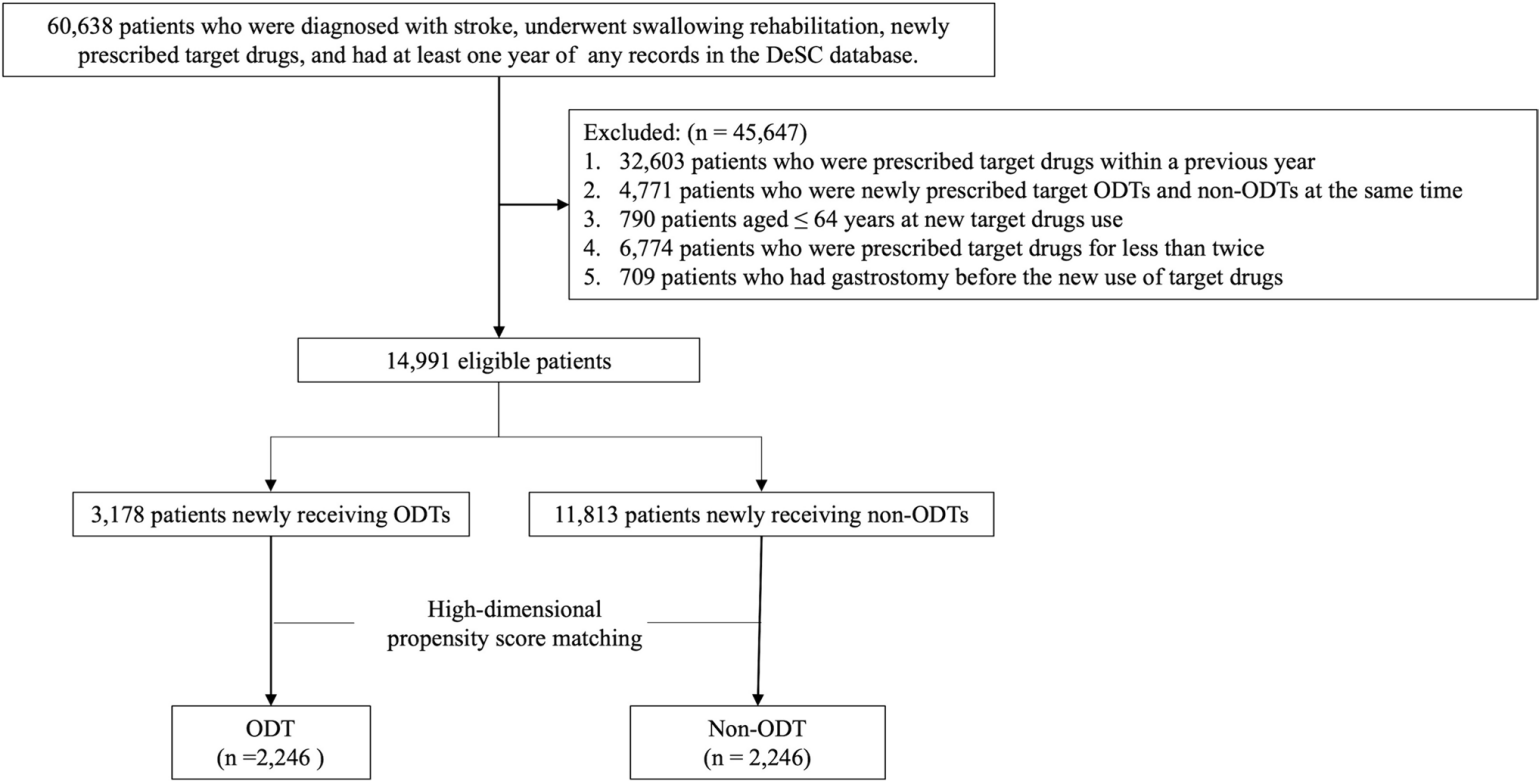
Flowchart of the selection of study participants. ODTs, orally disintegrating tablets

After applying the inclusion and exclusion criteria, 14,991 patients were identified between April 2014 and March 2020 (Fig. 1). Of those, 10,376 (69%) received antihypertensive drugs, 1844 (12%) received antidiabetic drugs, and 2771 (18%) received antidyslipidemic drugs. Propensity score matching created 2246 pairs.

Table 1 shows the baseline characteristics before and after propensity score matching. Before matching, the non-ODT group had a higher proportion of patients with diabetes, myocardial infarction, angina pectoris, atrial fibrillation, heart failure, asthma, gastroesophageal reflux disease, and renal failure but a lower proportion of patients with dementia, hypertension, and patients who underwent nasal feeding. Patient characteristics were balanced between the groups after propensity score matching.

**Table 1:** Baseline characteristics before and after 1:1 high-dimensional propensity score matching

	Unmatched groups				ASD	High-dimensional propensity score-matched groups				ASD
	Non-ODT		ODT			Non-ODT		ODT		
	n = 11,813	(%)	n = 3178	(%)		n = 2246	(%)	n = 2246	(%)	
Age										
65–74	1546	(13)	368	(12)	4.6	279	(12)	271	(12)	1.1
≥ 75	10,267	(87)	2810	(88)	4.6	1967	(88)	1975	(88)	1.1
Male	6406	(54)	1564	(49)	10.0	1112	(50)	1137	(51)	2.2
Human immunodeficiency virus	5	0	1	0	0.0	1	(0)	0	0	0.0
Sarcoidosis	26	(0)	1	0	6.3	1	(0)	1	(0)	0.0
Diabetes	6969	(59)	1333	(42)	34.7	973	(43)	986	(44)	1.2
Hypoglycemia	400	(3)	60	(2)	9.3	45	(2)	53	(2)	2.5
Amyloidosis	73	(1)	16	(1)	1.4	13	(1)	11	(0)	1.2
Dementia	3876	(33)	1210	(38)	11.1	819	(36)	819	(36)	0.0
Parkinson’s disease or parkinsonian disorder	1203	(10)	325	(10)	0.0	215	(10)	226	(10)	1.6
Hypertension	10,830	(92)	3021	(95)	13.7	2122	(94)	2112	(94)	1.9
Myocardial infarction	946	(8)	131	(4)	16.4	104	(5)	103	(5)	0.2
Angina pectoris	4938	(42)	910	(29)	27.9	679	(30)	691	(31)	1.2
Atrial fibrillation	4211	(36)	679	(21)	31.9	511	(23)	507	(23)	0.4
Heart failure	8423	(71)	1861	(59)	26.9	1328	(59)	1341	(60)	1.2
Cerebrovascular disease	11,813	(100)	3178	(100)	0.0	2246	(100)	2246	(100)	0.0
Aspiration pneumonia	4580	(39)	1358	(43)	7.9	1043	(46)	1086	(48)	3.8
Other pneumonia	5880	(50)	1558	(49)	1.6	912	(41)	903	(40)	0.8
COPD	810	(7)	206	(7)	1.6	136	(6)	133	(6)	0.6
Asthma	2656	(23)	579	(18)	10.7	425	(19)	428	(19)	0.4
Gastroesophageal reflux disease	8779	(74)	2142	(67)	15.2	1527	(68)	1524	(68)	0.3
Achalasia	197	(2)	48	(2)	1.6	42	(2)	35	(2)	2.4
Disorder after digestive system treatment	164	(1)	48	(2)	0.8	30	(1)	33	(1)	1.1
Scleroderma	46	(0)	8	(0)	1.7	3	(0)	5	(0)	0.0
Sjögren’s syndrome	410	(4)	91	(3)	3.4	60	(3)	68	(3)	2.0
Renal failure	3103	(26)	618	(19)	16.5	505	(22)	469	(21)	3.9
Other specified symptoms and signs involving the digestive system and abdomen	2330	(20)	748	(24)	9.2	484	(22)	481	(21)	1.3
Antiepileptic drugs	3325	(28)	925	(29)	2.2	608	(27)	619	(28)	1.1
Parkinson’s disease drugs	174	(2)	45	(1)	0.8	29	(1)	30	(1)	0.4
Hypnotic	7857	(67)	1968	(62)	9.6	1417	(63)	1396	(62)	2.1
Rehabilitation for cerebrovascular diseases	7350	(62)	2101	(66)	8.1	1484	(66)	1462	(65)	2.1
Nasal feeding	2827	(24)	1005	(32)	17.3	616	(27)	601	(27)	1.5
Tracheotomy	160	(1)	51	(2)	1.6	32	(1)	33	(1)	4.3
Oral care for preoperative periods	439	(4)	119	(4)	0.0	82	(4)	75	(3)	1.7

*ASD* absolute standardized difference, *COPD* chronic obstructive pulmonary disease, *ODT* orally disintegrating tablet

The primary outcomes before and after propensity score matching are shown in Table 2. The PDC exceeded 80% for the two groups before and after matching. There was no significant difference between the non-ODT and ODT group before (0.887 vs. 0.900, P = 0.999) or after (0.889 vs. 0.902, P = 0.977) propensity score.

**Table 2:** The proportion of days covered for one year, and the proportion of admission for cardiovascular events and aspiration pneumonia between non-ODT and ODT groups before and after high-dimensional propensity score-matching

	Unmatched groups		P value	High-dimensional propensity score-matched groups		P value
	Non-ODT	ODT		Non-ODT	ODT	
Primary outcome						
PDC for 1 year	0.887	0.900	0.999	0.889	0.902	0.977
Secondary outcome						
Heart failure	0.529	0.391	< 0.001	0.409	0.403	0.715
Atrial fibrillation	0.312	0.180	< 0.001	0.195	0.193	0.850
Myocardial infarction	0.045	0.025	< 0.001	0.023	0.028	0.299
Angina pectoris	0.261	0.171	< 0.001	0.183	0.183	0.969
Stroke	0.771	0.812	< 0.001	0.810	0.798	0.310
Composite event of cardiovascular diseases	0.904	0.903	0.778	0.898	0.893	0.591
Aspiration pneumonia	0.363	0.400	< 0.001	0.380	0.372	0.558

Composite event of cardiovascular diseases consists of heart failure, atrial fibrillation, myocardial infarction, angina pectoris, and stroke

*ODT* orally disintegrating tablet, *PDC* proportion of days covered

The secondary outcomes are presented in Table 2. Before propensity score matching, the non-ODT group had a significantly higher proportion of heart failure, atrial fibrillation, myocardial infarction, and angina pectoris than the ODT group, and a significantly lower proportion of stroke and aspiration pneumonia than the ODT group. However, no significant differences were observed between the non-ODT and ODT groups after propensity score matching.

Online Resource 4 shows the results of the subgroup analyses. None of the subgroups showed significant differences in primary outcomes between the two groups. Regarding secondary outcomes, none of the subgroups showed significant differences between the two groups, except for myocardial infarction in the antidyslipidemic drug subgroup.

## Discussion/Conclusion

We compared medication adherence in patients with dysphagia who received non-ODTs and ODTs, using a large commercial medical and dental claims database. There was no significant difference between the non-ODT and ODT groups in terms of the PDC. In addition, there was no significant difference in the occurrence of cardiovascular events and aspiration pneumonia within 1 year between the two groups.

Previous studies have suggested that because patients with dysphagia can take ODTs more easily than non-ODTs, their PDC improves [1, 5, 7]. However, our results did not show a significant difference between the two groups in terms of the PDC. This may be because the Japanese patients adhered well to their medications. A previous study showed higher medication adherence among Japanese patients with ulcerative colitis than among their European counterparts [22]. This could be attributed to the tendency of Japanese patients to follow instructions diligently [23]. In addition, one-third of the patients with hypertension, diabetes, and dyslipidemia in the United States were reported to be < 80% in terms of PDC [24]. However, this differs from the higher rates observed in this study. The current study showed a PDC comparable to that in previous Japanese studies on medication adherence in hypertension [25], diabetes [26], and dyslipidemia [27]. Specifically, medication adherence was good, with the majority of patients achieving a PDC > 80%. Although there was an overall trend toward lower medication adherence for polypharmacy, there was no significant difference in medication adherence between the ODT and non-ODT groups. There were also no significant differences in medication adherence for drug-targeted diseases. Given that ODTs are typically larger in size than non-ODTs and cause taste-related problems [28], clinicians may prescribe ODTs or non-ODTs based on patient preferences rather than only on post-stroke conditions.

No significant differences were observed in the incidence of aspiration pneumonia. The results of the current study are similar to those of a previous study, which showed no difference in the proportion of aspiration between ODTs and non-ODTs [5, 29]. Therefore, clinicians may not consider the increased risk of aspiration when prescribing non-ODTs.

This study has several limitations. First, this was a retrospective study that used a large commercial medical and dental claims database. Therefore, information and selection biases may have affected the results. To reduce this bias, we performed hd-PS matching. However, unmeasured confounding factors may have affected these results. Second, medication adherence was assessed using a claims database. However, it is unclear whether this accurately reflects actual medication adherence, as we were unable to observe when the patients took their medications. Nevertheless, this study followed the methods recommended by the National Association of Specialty Pharmacy Clinical Outcomes Committee Adherence Workgroup and the American Society of Health-System Pharmacists Section of Specialty Pharmacy Practitioners Outcomes and Value Section Advisory Group [15], which ensured the validity of our results.

In conclusion, there was no difference in medication adherence between the ODT and the non-ODT groups of patients with post-stroke dysphagia, and both groups had a good PDC exceeding 80%. Clinicians may consider prescribing ODTs or non-ODTs based on patient preferences rather than solely on post-stroke conditions. Therefore, further studies on other etiologies of dysphagia, such as sarcopenia, are warranted.

## Supplementary Information

Below is the link to the electronic supplementary material.

Supplementary file1 (DOCX 44 kb)

## Data Availability

The datasets analyzed in the current study are commercially available, DeSC Healthcare, Inc. providing the database.
