# Supplementary material for: Do Orally Disintegrating Tablets Facilitate Medical Adherence and Clinical Outcomes in Patients with Post-stroke Dysphagia?
Source: Dysphagia. 2024 Aug 3;40(2):381–7. doi: 10.1007/s00455-024-10737-8 (PMC11893695; doi:10.1007/s00455-024-10737-8)
Supplement: Supplementary file 1 — Supplementary file1 (DOCX 44 kb) [file 455_2024_10737_MOESM1_ESM.docx]

**Do orally disintegrating tablets facilitate medical adherence and clinical outcomes in patients with post-stroke dysphagia?**

So Sato,^a^ Yusuke Sasabuchi ,^a, b^ Akira Okada,^c^ Hideo Yasunaga^a^

**Online Resource 1.** The detail of the target orally disintegrating tablets

**Online Resource 2.** Covariates used for estimation of propensity scores

**Online Resource 3.** Oral medication classes extracted in this study

**Online Resource 4.** The proportion of days covered for one year, and the proportion of admissions for cardiovascular events and aspiration pneumonia between the non-ODT and ODT groups before and after high-dimensional propensity score matching (subgroup analysis)

**Online Resource 1.** The detail of the target orally disintegrating tablets

| Antihypertensive drugs | Antidiabetic drugs | Antidyslipidemic drugs |
| --- | --- | --- |
| Amlodipine besylate  Azilsartan/amlodipine besylate combination  Candesartan cilexetil  Doxazosin mesylate  Irbesartan  Olmesartan medoxomil  Telmisartan  Valsartan  Valsartan/amlodipine besylate combination | Acarbose  Glimepiride  Luseogliflozin hydrate  Miglitol  Mitiglinide calcium hydrate  Mitiglinide calcium hydrate/voglibose combination  Pioglitazone hydrochloride  Teneligliptin hydrobromide hydrate  Voglibose | Atorvastatin calcium hydrate  Ezetimibe  Pitavastatin calcium  Rosuvastatin calcium |

**Online Resource 2.** Covariates used for estimation of propensity scores

| Patient characteristic | Comorbidity (ICD-10 codes) | Drug (ATC classification) | Intervention (Japanese procedure code) |
| --- | --- | --- | --- |
| Age  Sex | Human immunodeficiency virus infection (B20–B24)  Sarcoidosis (D86)  Diabetes (E10–E14)  Hypoglycemia (E15–E16)  Amyloidosis (E85)  Dementia (F00–F02)  Parkinson’s disease or parkinsonian disorder (G20–G22)  Hypertension (I10, I15)  Myocardial infarction (I21)  Angina pectoris (I20)Atrial fibrillation (I48)  Heart failure (I50, I11)  Stroke (I60–I63)  Aspiration pneumonia (J69)  Other Pneumonia (J12–18, J84)  Chronic obstructive pulmonary disease (J44)  Asthma (J45)  Gastroesophageal reflux disease (K21)  Postprocedural disorders of digestive system (K91)  Scleroderma (L94, M34)  Sjögren's syndrome (M35)  Renal failure (N17–N19)  Other specified symptoms and signs involving the digestive system and abdomen (R19.8) | Antiepileptic drugs (N03)  Parkinson’s disease drugs and anticholinergic drugs (N04A)  Hypnotics (N05C) | Cerebrovascular rehabilitation fee (H001)  Nasal feeding (J120)  Tracheotomy (K386)  Perioperative oral care fee (B000-5–B000-8) |

ICD, International Classification of Diseases and Related Health Problems, 10th revision code; ATC, Anatomical therapeutic chemical

**Online Resource 3**. Oral medication classes extracted in this study

| Category | Class of medication |
| --- | --- |
| Antipsychotic drugs and hypnotics | Benzodiazepine anxiolytic |
|  | Ultrashort-acting benzodiazepine hypnotic |
|  | Short-acting benzodiazepine hypnotic |
|  | Middle- to long-acting benzodiazepine hypnotic |
|  | Ultrashort-acting non-benzodiazepine hypnotic |
|  | Barbiturate |
|  | Diazepam |
|  | Melatonin receptor agonist |
|  | Orexin receptor antagonist |
|  | Benzamide |
|  | Butyrophenone |
|  | Phenothiazine |
|  | Serotonin dopamine antagonist |
|  | Multi-acting receptor-targeted antipsychotic |
|  | Dopamine system stabiliser |
|  | Dopamine partial agonist |
|  | Lithium carbonate |
|  | 5-HT1A agonist |
|  | Tricyclic antidepressant |
|  | Tetracyclic antidepressant |
|  | Selective serotonin reuptake inhibitor |
|  | Serotonin noradrenaline reuptake inhibitor |
|  | Noradrenergic and specific serotonergic antidepressant |
|  | Others |
| Antidementia drugs | Donepezil |
|  | Galantamine |
|  | Memantine |
| Anticoagulants and antiplatelets | Antiplatelet |
|  | Anticoagulant |
|  | Direct oral anticoagulant |
| Hypotensive drugs and diuretics | Ca2+ blocker (excluding verapamil) |
|  | Angiotensin II receptor blocker |
|  | Angiotensin-converting enzyme inhibitor |
|  | α-blocker |
|  | β-blocker |
|  | Sympatholytic |
|  | Loop diuretic |
|  | Spironolactone |
|  | Thiazide |
|  | Others, including combination drugs |
| Antiarrhythmic drugs | Class 1a |
|  | Class 1b |
|  | Class 1c |
|  | Class 3 |
|  | Class 4 |
|  | Digoxin |
|  | Verapamil |
| Hyperlipemia medicines | Statin |
|  | Fibrate |
|  | Ezetimibe |
|  | Eicosapentaenoic acid |
|  | Nicotinic acid |
|  | Others, including combination drugs |
| Gastrointestinal drugs | Proton pump inhibitor |
|  | Potassium-competitive acid blocker |
|  | H2 antagonist |
|  | Muscarinic antagonist |
|  | Prostaglandin analogue |
|  | Sucralfate |
|  | Others, including combination drugs |
| Diabetes therapeutic drugs | Biguanide |
|  | Thiazolidine |
|  | Dipeptidyl peptide-4 inhibitor |
|  | Sulfonylurea |
|  | Glinide |
|  | α-glucosidase inhibitor |
|  | Sodium-glucose cotransporter 2 inhibitor |
|  | Others, including combination drugs |
| Analgesics | Acetaminophen |
|  | Non-steroidal anti-inflammatory drug |
|  | Cyclooxygenase inhibitor |
|  | Pregabalin |
|  | Tramadol |
|  | Others, including combination drugs |
| Japanese herbal medicines | All herbal medicines prescribed |
| Enteral nutrition | All enteral nutrition (drugs only) |
| Other drugs | Anticholinergic drug |
|  | L-Dopa |
|  | α-blocker |
|  | 5α-reductase inhibitor |
|  | Oxybutynin |
|  | Muscarinic antagonist |
|  | α-blocker |
|  | Antiemetic |
|  | Prochlorperazine maleate |
|  | H1 antagonist |

**Online Resource 4.** The proportion of days covered for one year, and the proportion of admissions for cardiovascular events and aspiration pneumonia between the non-ODT and ODT groups before and after high-dimensional propensity score matching (subgroup analysis)

|  | Unmatched groups | |  | High-dimensional propensity score-matched groups | |  |
| --- | --- | --- | --- | --- | --- | --- |
|  |  |  | p value |  |  | p value |
|  | non-ODTs | ODTs |  | non-ODTs | ODTs |  |
| Primary outcome | |  |  |  |  |  |
| No polypharmacy | 0.904 | 0.923 | 0.949 | 0.912 | 0.921 | 0.814 |
| Polypharmacy | 0.894 | 0.901 | 0.832 | 0.886 | 0.907 | 0.981 |
| Hyperpolypharmacy | 0.882 | 0.888 | 0.816 | 0.888 | 0.889 | 0.564 |
| Antihypertensive drugs | 0.887 | 0.899 | 0.994 | 0.889 | 0.902 | 0.966 |
| Antidiabetic drugs | 0.887 | 0.883 | 0.463 | 0.874 | 0.883 | 0.568 |
| Antidyslipidemic drugs | 0.888 | 0.908 | 0.957 | 0.895 | 0.905 | 0.764 |
| Secondary outcome | |  |  |  |  |  |
| Antihypertensive drugs | | |  |  |  |  |
| Heart failure | 0.581 | 0.399 | <0.001 | 0.428 | 0.418 | 0.524 |
| Atrial fibrillation | 0.356 | 0.178 | <0.001 | 0.200 | 0.193 | 0.557 |
| Myocardial infarction | 0.046 | 0.022 | <0.001 | 0.024 | 0.023 | 0.902 |
| Angina pectoris | 0.273 | 0.172 | <0.001 | 0.193 | 0.189 | 0.760 |
| Stroke | 0.764 | 0.804 | <0.001 | 0.804 | 0.785 | 0.160 |
| Composite event of cardiovascular diseases | 0.913 | 0.899 | 0.031 | 0.897 | 0.886 | 0.291 |
| Aspiration pneumonia | 0.373 | 0.413 | <0.001 | 0.385 | 0.385 | 0.995 |
| Antidiabetic drugs | |  |  |  |  |  |
| Heart failure | 0.475 | 0.423 | 0.601 | 0.416 | 0.423 | 0.947 |
| Atrial fibrillation | 0.233 | 0.077 | 0.060 | 0.195 | 0.077 | 0.152 |
| Myocardial infarction | 0.048 | 0.039 | 0.823 | 0.013 | 0.039 | 0.416 |
| Angina pectoris | 0.27 | 0.077 | 0.027 | 0.169 | 0.077 | 0.251 |
| Stroke | 0.763 | 0.731 | 0.702 | 0.740 | 0.731 | 0.924 |
| Composite event of cardiovascular diseases | 0.886 | 0.846 | 0.531 | 0.870 | 0.846 | 0.758 |
| Aspiration pneumonia | 0.391 | 0.423 | 0.736 | 0.494 | 0.423 | 0.534 |
| Antidyslipidemic drugs | | |  |  |  |  |
| Heart failure | 0.404 | 0.333 | 0.007 | 0.334 | 0.332 | 0.939 |
| Atrial fibrillation | 0.23 | 0.201 | 0.206 | 0.175 | 0.202 | 0.316 |
| Myocardial infarction | 0.037 | 0.049 | 0.235 | 0.022 | 0.051 | 0.024 |
| Angina pectoris | 0.215 | 0.165 | 0.025 | 0.148 | 0.164 | 0.524 |
| Stroke | 0.798 | 0.871 | 0.001 | 0.845 | 0.868 | 0.355 |
| Composite event of cardiovascular diseases | 0.893 | 0.936 | 0.010 | 0.907 | 0.933 | 0.183 |
| Aspiration pneumonia | 0.31 | 0.307 | 0.893 | 0.343 | 0.302 | 0.211 |

No polypharmacy: 0–4 prescription, polypharmacy: 5–9 prescription, hyper-polypharmacy: ≥10 prescription. Composite event of cardiovascular diseases consists of heart failure, atrial fibrillation, myocardial infarction, angina pectoris, and stroke. Abbreviations: ODT, orally disintegrating tablet; PDC, proportion of days covered.
